# The oldest record of aquatic amniote congenital scoliosis

**DOI:** 10.1371/journal.pone.0185338

**Published:** 2017-09-21

**Authors:** Tomasz Szczygielski, Dawid Surmik, Agnieszka Kapuścińska, Bruce M. Rothschild

**Affiliations:** 1 Institute of Paleobiology, Polish Academy of Sciences, Warsaw, Poland; 2 Park of Science & Human Evolution, Krasiejów, Poland; 3 Faculty of Earth Sciences, University of Silesia, Sosnowiec, Poland; 4 Carnegie Museum, Pittsburgh, Pennsylvania, United States of America; 5 West Virginia University School of Medicine, Morgantown, West Virginia, United States of America; Institute of Botany, CHINA

## Abstract

We report the first occurrence of congenital scoliosis in an early Permian aquatic parareptile, *Stereosternum tumidum* from Paraná state, Brazil. The spine malformation is caused by a congenital hemivertebra. These observations give insight into the biomechanical aspects of underwater locomotion in an axial skeleton-compromised aquatic amniote. This is the oldest record of a hemivertebra in an aquatic animal.

## Introduction

Mesosaurs are early Permian aquatic amniotes, nested at the base of the Parareptilia[1–4]. They include three monotypic genera (*Brazilosaurus sanpauloensis* Shikama [5], *Mesosaurus tenuidens* Gervais [6], and *Stereosternum tumidum* Cope [7]) known from South America and Africa [5–15].

Hemivertebra is a congenital malformation in vertebral structure, formed as a result of failure of development of one side of a vertebra and results from a segmentation defect during osteogenesis [16,17] or as an effect of fusion of contralateral vertebral anlages belonging to different (mismatched) body segments (hemimetameric segemental shift) [18]. The causes are unknown. Numerous factors may come into play, including chromosome 7q36 deletion in humans [19]. The occurrence of hemivertebra frequently results in angulation of the vertebral column and thus produces kyphosis, scoliosis and/or lordosis [17,20], although in some cases the adjacent vertebrae change their shape to compensate and minimalize the curvature of the vertebral column (incarcerated hemivertebra [16]). Hemivertebrae have been previously described in a number of amphibian, reptilian and mammalian taxa. They have, however, a scarce record in the Paleozoic, represented mostly in Permian temnospondyls [17]. The only amniotic case of hemivertebra reported thus far was in an early Permian (Artinskian, according to Holterhoff et al. [21]) captorhinomorph [22]. See the S1 File and Rothschild et al. [23] for more recent occurrences.

### Institutional abbreviation

**ZPAL**, Institute of Paleobiology, Polish Academy of Sciences, Warsaw, Poland.

## Materials and methods

**Fig 1:**
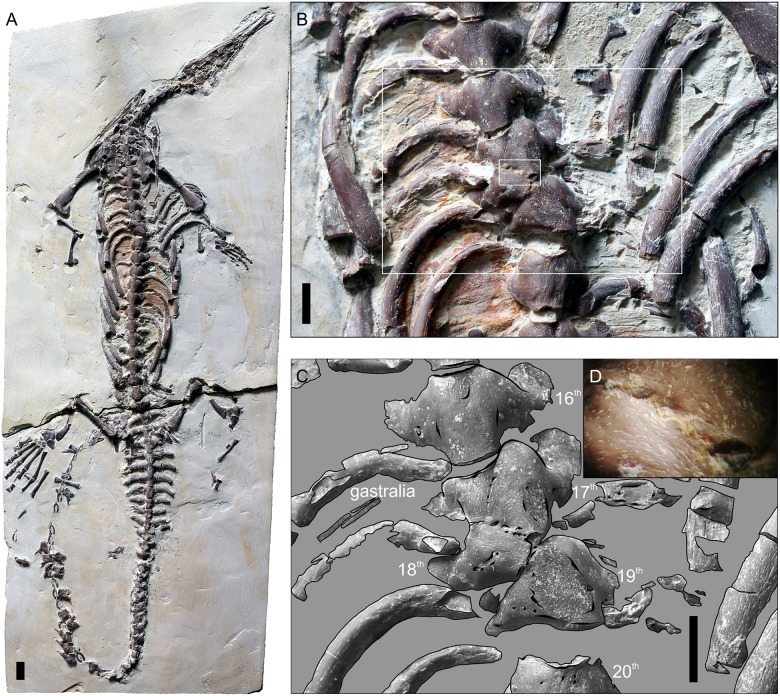
*Stereosternum tumidum* specimen ZPAL R VII/1. (A) View of the complete specimen. (B) Enlarged ribcage area showing 16^th^ to 20^th^ dorsal vertebra with hemivertebra (18^th^ dorsal vertebra). The larger rectangle indicates the area shown in C and the smaller one the area shown in D. (C) Outlines of bones in marked region of B. (D) Close-up of the contact between the hemivertebra and the preceding vertebra. All scale bars equal 1 cm, D not to scale.

ZPAL R VII/1, a complete mesosaur skeleton (Fig 1A), was examined. The specimen came from the Paraná state, Brazil, bought in the mid-1990s from a private collector, Zofia Zarańska. Initially identified as *Mesosaurus brasiliensis* and dated to the Late Permian, it was later reassessed by Kapuścińska [24] as *Stereosternum tumidum* and dated to the Early Permian. The specimen is a nearly complete, mostly articulated, moderately compacted skeleton (total length approx. 80 cm), embedded in a laminated, pyroclastic mudstone (most likely tonstein, according to Kapuścińska [24]) and exposed in ventral view. Vertebral malformation was briefly described and illustrated by Kapuścińska [24], without recognition of the specific diagnosis. The description provided by Kapuścińska was a part of her Master’s thesis, and never formally published.

## Results

### Systematic paleontology

Parareptilia Olson, 1947 [25]

Mesosauridae Baur, 1889 [26]

*Stereosternum tumidum* Cope, 1886 [7]

### Geographic and stratigraphic range

South America (south-eastern Brazil, north-western and central Uruguay, western Paraguay, north-western Argentina) and northern Africa (western and central RSA, southern and north-western Namibia). *Stereosternum tumidum* remains from Paraná, Brazil were described from lower Permian (Artinskian) Iraty Shale Formation [1,2], the age according to U-Pb zircon dating, see Santos et al. [27].

### Description of the specimen

The vertebrae and ribs are pachyostotic. A right-sided hemivertebra (Fig 1B and 1C) is present at the anterior dorsal level (between the 17^th^ and 19^th^ dorsal vertebrae). It is trapezoid in shape in ventral view. This right-sided hemivertebra produced opposite deviation of the subsequent complete vertebra and compensatory separation (apparent disk space increase) from the succeeding vertebra. The rib associated with the hemivertebra is angulated proximally and slightly thinner than ribs on adjacent vertebra. The missing vertebral segment lacks a rib. S-shaped scoliosis was produced as an effect of accommodation of the shape of adjacent vertebrae in order to restore the longitudinal orientation of the vertebral column. Therefore, the hemivertebra may be classified as incarcerated. The hemivertebra is fused to the preceding centrum, but the boundaries between each element are readily visible. The contact between them is suture-like (Fig 1D) and notably differs from the normal vertebral limits visible in that specimen. The presence of only a single hemivertebra preceded and followed by normally developed vertebrae indicates that this is not the case of a hemimetameric vertebral shift, but rather of a failure of development of the left vertebral anlage.

The 17^th^ vertebra has an asymmetrically developed transverse processes: the right one is atrophied, while the left one is well-developed. Similar asymmetry, although weaker, is also visible for the 19^th^ vertebra. Their centra are likewise asymmetrical, and their ventral surfaces appear to be slightly skewed to the left (it is unsure whether they retained this position in life, or if it results from their post-mortem disarticulation). The ventral surfaces (especially of the 19^th^ vertebral centrum) also appear to be anomalously flattened and are more roughly-textured than in the other vertebrae.

## Discussion

The distortion of the axial skeleton found in several specimens of mesosaurs is caused by post-mortem processes, such as disarticulation and displacement of elements caused by scavengers or decomposition or tectonic folding of the rock matrix. In these cases, one of the vertebrae may be pushed into the matrix and only partially exposed as a wedge-shaped element between the transverse processes of adjacent vertebrae. The condition in ZPAL R VII/1, however, clearly occurred in vivo. First of all, the contact between the hemivertebra and the preceding centrum undoubtedly is not an effect of disarticulation and relative dislocation of these elements. The surface of their contact is not sharp and straight, as it would be if these elements were separate in life, but undulates in a zig-zag manner and bears miniscule pits, the layout of which agrees closely on both elements (Fig 1D). They are located at the same level, neither is pushed dorsally, and their ventral surfaces are confluent (S1 File). The shape of the adjacent vertebrae is modified to accommodate the hemivertebra. There is no space between the 17^th^ and 19^th^ vertebra that would fit the remaining part of the 18^th^ vertebra, if it was complete. Additionally, the rib of the hemivertebra is slightly atrophied. Neither of these changes can be explained as an effect of disarticulation or tectonic processes, as they would only impact the layout of the bones, not their shape. Even strong compaction would be unlikely to cause such an ideal alignment of these pachyostotic vertebrae, and it would certainly leave evidence on surrounding bones, which are not strongly compacted in ZPAL R VII/1. Additionally, tectonic processes may be excluded due to the focal character of the condition observed in ZPAL R VII/1 –there is no evidence of such processes anywhere in the limbs, vertebrae, or matrix grain around the hemivertebra.

The presence of hemivertebra may compromise neurologic function, producing rear-limb weakness and paralysis [28,29]. The mid-column localization of the mesosaur’s vertebral malformation likely reduced its flexibility, but apparently, the malformation had no noticeable negative influence on the animal’s locomotion and hunting ability, which would likely had resulted in its death at a young age. Based on the large size (compare to Rossmann [10,14]), the absence of the suture which divides the procoracoid and metacoracoid in juveniles [30] and the presence of a well-ossified tarsus [31], the specimen is considered an adult [24]. This supports the view that lateral undulations of the trunk were not the key factor of locomotion in mesosaurs [2,24,32–35]. Possibly, their slow-paced swimming technique required only minor trunk flexibility or they mainly utilized the posterior portion of the vertebral column, using mostly the tail-driven propulsion, in a sub-carangiform (sub-anguilliform, axial subundulatory) fashion [2,24,32,34,35]. This agrees with the speculation as to feeding habits of mesosaurs, which were shown to prey mostly on small, slow-moving pygocephalomorph crustaceans and occasionally scavenged on mesosaurid corpses [9,34,36,37].

## Conclusions

Our study presents one of the very few cases of fossil hemivertebra in a complete, articulated skeleton (usually only a small, isolated fragment of vertebral column including the hemivertebra and neighboring vertebrae is found), which illustrates the effect of malformation on the whole body of the animal. Additionally, this is the oldest evidence of congenital scoliosis in aquatic amniote, dated back to early Permian. Although it seems to be more or less contemporary with reported occurrence of hemivertebra in a Permian captorhinomorph [22], the latter was neither illustrated nor described in detail. The case presented here documents persistence of homeobox defects and the apparent importance of restoration of normal anteroposterior organization of the axial skeleton, even in an aquatic medium, thus lowering the mechanical loads imposed on the vertebral column. In animals with weight-bearing spinal columns, such accommodations are necessary to compensate for postural deviation. This seems the first documentation in a non-weight-bearing vertebral column.

## Supporting information

S1 File Expanded list of occurrences of congenital scoliosis and additional photographs of *Stereosternum tumidum* ZPAL R VII/1 specimen. (PDF)
