# Supplementary material for: The oldest record of aquatic amniote congenital scoliosis
Source: PLoS One. 2017 Sep 21;12(9):e0185338. doi: 10.1371/journal.pone.0185338 (PMC5608408; doi:10.1371/journal.pone.0185338)
Supplement: S1 File — (PDF) [file pone.0185338.s001.pdf]

THE OLDEST RECORD OF AQUATIC AMNIOTE CONGENITAL SCOLIOSIS

SUPPLEMENTARY INFORMATION:

EXTENDED DATA

AND

DISCUSSION

TOMASZ SZCZYGIELSKI[1,*], DAWID SURMIK[2,3], AGNIESZKA KAPUŚCIŃSKA[1], AND BRUCE M. ROTHSCHILD[4,5]

[1]Institute of Paleobiology, Polish Academy of Sciences, Twarda 51/55, 00-818 Warsaw, Poland
[2]Park of Science & Human Evolution, 1 Maja 10, 46-040 Krasiejów, Poland
[3]Faculty of Earth Science, University of Silesia, Będzińska 60, 41-200 Sosnowiec, Poland
[4]Carnegie Museum, 4400 Forbes Ave, Pittsburgh, Pennsylvania, 15213 U.S.A.
[5]West Virginia University School of Medicine, Morgantown, West Virginia, 26506 U.S.A.

*E-mail: t.szczygielski@twarda.pan.pl

BIBLIOGRAPHY ON HEMIVERTEBRAE

Hemivertebrae have been previously described in a number of taxa spanning from the Paleozoic to Recent. Occurrences in amphibians were thus far described in Permian (Witzmann et al., 2014) and Triassic (Witzmann, 2007; Witzmann et al., 2014) temnospondyls as well as in modern taxa (Raichoudhury and Das, 1931; Pawłowska-Indyk, 1976; Martínez et al., 1992; Perpiñán et al., 2010). In reptiles, they were found in a Permian captorhinomorph (Johnson, 1988), a Jurassic ornithopod dinosaur (Witzmann et al., 2008), and a Jurassic plesiosaur (Lydekker, 1889), and in modern taxa (Albrecht, 1883; Baur, 1891). The record of hemivertebrae in mammals begins with an Oligocene nimravid (Burnham et al., 2013) and is pretty well represented in modern taxa, mostly in medical and veterinary literature (e.g., Done et al., 1975; Shawen et al., 2002; Besalti et al., 2005; Kawakami et al., 2009; Schlensker and Distl, 2013). See Rothschild et al. (2012) for an exhaustive list of osteopatologies in amphibians and reptiles, including the occurences of hemivertebrae.

REFERENCES

1. Albrecht, P. 1883. Une hémivertébre gauche surnuméraire, de *Python sebae*, Duméril. Bulletin Du Musée Royal d'Histoire Naturelle de Belgique 2:21–34.
2. Baur, G. 1891. On intercalation of vertebrae. Journal of Morphology 4:331–336.
3. Besalti, O., A. Ozak, Z. Pekcan, and S. Eminaga. 2005. Nasca classification of hemivertebra in five dogs. Irish Veterinary Journal 58:688–690.
4. Burnham, D. A., B. M. Rothschild, J. P. Babiarz, and L. D. Martin. 2013. Hemivertebrae as pathology and as a window to behavior in the fossil record. PalArch's Journal of Vertebrate Palaeontology 10:1–6.
5. Done, S. H., R. A. Drew, G. M. Robins, and J. G. Lane. 1975. Hemivertebra in the dog: Clinical and pathological observations. Veterinary Record 96:313–317.
6. Imagama, S., N. Kawakami, and N. Ishiguro. 2011. Three-dimensional CT analysis of congenital scoliosis and kyphosis: A new classification. In: Subburaj, K. (ed.) CT Scanning - Techniques and Applications. InTech, 358 pp.
7. Johnson, G. D. 1988. Abnormal captorhinomorph vertebra from the lower Permian of north-central Texas. Journal of Vertebrate Paleontology 8:19A.
8. Kawakami, N., T. Tsuji, S. Imagama, L. G. Lenke, R. M. Puno, T. R. Kuklo, and Spinal Deformity Study Group. 2009. Classification of congenital scoliosis and kyphosis, a new approach to the three-dimensional classification for progressive vertebral anomalies requiring operative treatment. Spine 34:1756–1765.
9. Lydekker, R. 1889. Catalogue of the Fossil Reptilia and Amphibia in the British Museum (Natrual History) Part II. The Orders Ichthyopterygia and Sauropterygia. Order of the Trustees, London, 307 pp.
10. Martínez, I., R. Álvarez, I. Herráez, and P. Herráez. 1992. Skeletal malformations in hatchery reared *Rana perezi* tadpoles. The Anatomical Record 233:314–320.
11. Pawłowska-Indyk, A. 1976. Zmienność szkieletu osiowego kumaka górskiego (*Bombina varigata* L.) z okolic Krynicy (Beskid Sądecki) [Variability of the vertebral column of *Bombina varigata* (L.) of the Krynica region (Beskid Sądecki)]. Acta Universitatis Wratislaviensis. Prace Zoologiczne 7:57–68.
12. Perpiñán, D., M. M. Garner, J. G. Trupkiewicz, J. Malarchik, D. L. Armstrong, A. Lucio-Forster, and D. D. Bowman. 2010. Scoliosis in a tiger salamander (*Ambystoma tigrinum*) associated with encysted digenetic trematodes of the genus Clinostomum. Journal of Wildlife Diseases 46:579–584.
13. Raichoudhury, D. P., and B. K. Das. 1931. Observations on the malformations in the common Bengal toad, *Bufo melanosticus*. Anatomischer Anzeiger 71:120–131.
14. Rothschild, B. M., H.-P. Schultze, and R. Pellegrini. 2012. Herpetological Osteopathology. Annotated Bibliography of Amphibians and Reptiles. Springer Science+Business Media, New York, Dordrecht, Heidelberg & London, 450 pp.
15. Schlensker, E., and O. Distl. 2013. Prevalence, grading and genetics of hemivertebrae in dogs. European Journal Companion Animal Practice 23:119–123.
16. Shawen, S. B., P. J. Belmont, T. R. Kulko, B. D. Owens, K. F. Taylor, R. Kruse, and D. W. Polly. 2002. Hemimetameric segmental shift: a case series and review. Spine 15:E539–544.
17. Witzmann, F. 2007. A hemivertebra in a temnospondyl amphibian: the oldest record of scoliosis. Journal of Vertebrate Paleontology 27:1043–1046.
18. Witzmann, F., P. Asbach, K. Remes, O. Hampe, A. Hilger, and A. Paulke. 2008. Vertebral pathology in an ornithopod dinosaur: A hemivertebra in *Dysalotosaurus lettowvorbecki* from the Jurassic of Tanzania. Anatomical Record 291:1149–1155.
19. Witzmann, F., B. M. Rothschild, O. Hampe, G. Sobral, Y. M. Gubin, and P. Asbach. 2014. Congenital malformations of the vertebral volumn in ancient amphibians. Journal of Veterinary Medicine Series C: Anatomia Histologia Embryologia 43:90–102.

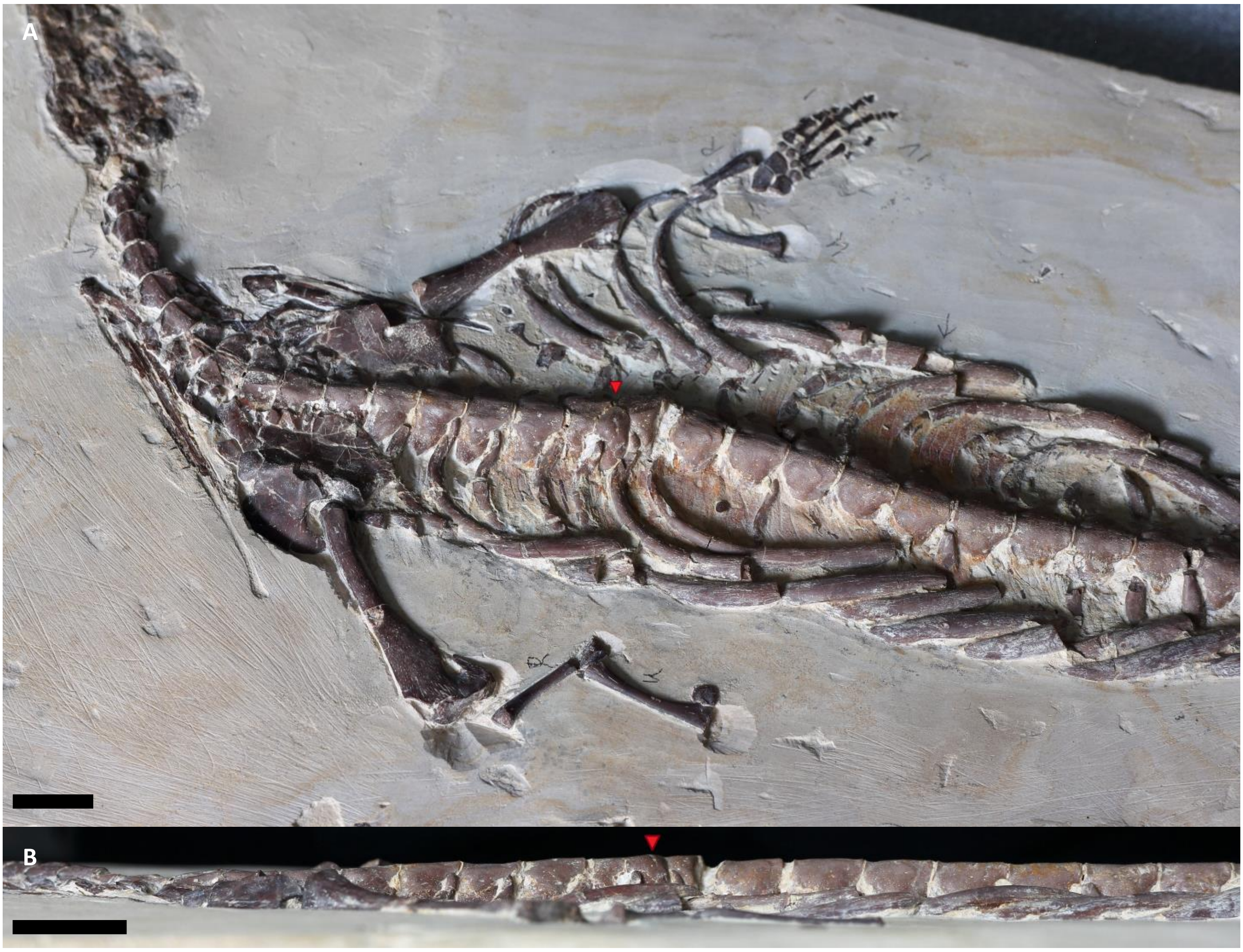

S1 Figure. *Stereosternum tumidum* ZPAL R VII/1. **A:** Lateroventral view. **B:** Lateral view (cranial to the left). Red arrowhead indicates the position of the hemivertebra. Note that the hemivertebra lies in the same plane as the surrounding vertebrae and shows no signs of relocation, compaction, or damage. Scale bars equal 1 cm.
